# Local immunotherapy with ONCOS-102 shapes harmful tumor associated CD68+ macrophages to become beneficial cells that correlate with increased overall survival

**DOI:** 10.1186/2051-1426-3-S2-O16

**Published:** 2015-11-04

**Authors:** Sari Pesonen, Johan Lundin, Nina Linder, Riku Turkki, Ari Ristimäki, Timo Joensuu, Kalevi Kairemo, Kaarina Partanen, Tuomo Alanko, Elke Jäger, Julia Karbach, Claudia Wahle, Akseli Hemminki, Charlotta Backman, Mikael von Euler, Tiina Hakonen, Tuuli Ranki, Antti Vuolanto, Magnus Jäderberg, Dmitry Zamarin

**Affiliations:** 1 Oncos Therapeutics, Helsinki, Finland; 2 Institute for Molecular Medicine Finland (FIMM), Helsinki, Finland; 3 Division of Pathology, HUSLAB and Hartman Institute, Helsinki University Central Hospital, Helsinki, Finland; 4 Docrates Cancer Center, Helsinki, Finland; 5 Hämatologie-Onkologie, Krankenhaus Nordwest, Frankfurt, Germany; 6 Cancer Gene Therapy Group, Medicum, Haartman Institute, University of Helsinki, and TILT Biotherapeutics Ltd, Helsinki, Finland; 7 Memorial Sloan Kettering Cancer Center, New York, NY, USA

## 

With the increasing excitement around new immunotherapeutic approaches, there has been a shift in the way viral cancer therapy is regarded from providing mainly oncolysis towards being a cancer immunotherapy. Adenoviruses have a unique ability to prime and boost immune responses. GM-CSF coding adenovirus ONCOS-102 causes immunogenic cancer cell death whereupon tumor antigens are presented into the immunogenic environment. ONCOS-102 has been previously shown to initiate CD8+ T cell responses against tumor-derived antigens in chemotherapy refractory cancer patients.

A total of 12 cancer patients were treated with repeated intratumoral injections of ONCOS-102 in a Phase I study. Sequential biopsies were collected at baseline and 1 and 2 months after treatment initiation and analyzed for the presence of immune cells by immunohistochemistry (IHC) in digitally scanned samples. Readout of the expression levels for innate immune cells (CD68, CD163, CD11c), T cells (CD3, CD4, CD8), and B cells (CD19) was performed in tumorous regions by the use of an image analysis algorithm based on color deconvolution and segmentation of the IHC stained cells. In an exploratory analysis, correlation between absolute expression levels of different immune cell markers in tumors and overall survival (OS) was assessed by Spearman´s rank correlation analysis.

At baseline, the absolute expression level of macrophage marker CD68 negatively correlated with OS (correlation coefficient (r)= -0.59, p=0.04), suggesting that tumor-associated macrophages (TAMs) in untreated tumors were tumorigenic. No correlation between other immune cell markers and OS was seen at baseline. 11/12 patients showed a post-treatment increase in tumor-infiltrating innate and adaptive immune cells, with the most prominent increase seen in CD8+ cells. In contrast to baseline, post-treatment samples showed a positive correlation between the expression level of CD68+ cells and OS (r=0.71, p=0.01), suggesting that CD68+ macrophages that were attracted into tumors after ONCOS-102 injection displayed different functionality than TAMs present prior to treatment. Furthermore, absolute expression levels of T cell markers CD3 (r=0.74, p=0.006), CD4 (r=0.76, p=0.004), and CD8 (r=0.73, p=0.007) in post-treatment biopsies all positively correlated with OS.

To summarize, ONCOS-102 induced infiltration of CD68+ macrophages and T cells which was associated with increased OS, while CD68+ TAMs pre-treatment was associated with shortened OS. This suggest that local immunotherapy with ONCOS-102 has the potential to activate immunologically silent tumors and reduce local immune suppression in advanced tumors.

